# Risk adjustment model for tuberculosis compared to non-tuberculosis mycobacterium or latent tuberculosis infection: Center for Personalized Precision Medicine of Tuberculosis (cPMTb) cohort database

**DOI:** 10.1186/s12890-023-02646-7

**Published:** 2023-11-24

**Authors:** Woo Jung Seo, Hyeon-Kyoung Koo, Ji Yeon Kang, Jieun Kang, So Hee Park, Hyung Koo Kang, Hye Kyeong Park, Sung-Soon Lee, Sangbong Choi, Tae Won Jang, Kyeong-Cheol Shin, Jee Youn Oh, Joon Young Choi, Jinsoo Min, Young-Kyung Choi, Jae-Gook Shin, Yong-Soon Cho

**Affiliations:** 1 Division of Pulmonary and Critical Care Medicine, Department of Internal Medicine, Ilsan Paik Hospital, Inje University College of Medicine, Goyang, Korea; 2 Division of Pulmonary and Critical Care Medicine, Department of Internal Medicine, Sanggye Paik Hospital, Inje University College of Medicine, Seoul, Korea; 3 Division of Pulmonary, Department of Internal Medicine, Kosin University College of Medicine, Kosin University Gospel Hospital, Busan, Korea; 4 Division of Pulmonology, Allergy and Critical Care Medicine, Department of Internal Medicine, College of Medicine, Yeungnam University, Yeungman University Medical Center, Daegu, Korea; 5 Division of Pulmonology, Allergy and Critical Care Medicine, Department of Internal Medicine, Korea University Guro Hospital, Korea University College of Medicine, Seoul, Korea; 6 Division of Pulmonary and Critical Care Medicine, Department of Internal Medicine, College of Medicine, Incheon St. Mary’s Hospital, The Catholic University of Korea, Incheon, Republic of Korea; 7 Division of Pulmonary and Critical Care Medicine, Department of Internal Medicine, College of Medicine, Seoul St. Mary’s Hospital, The Catholic University of Korea, Seoul, Korea; 8 Center for Personalized Precision Medicine of Tuberculosis (cPMTb), Inje University College of Medicine, Busan, 47392 Korea; 9 Department of Pharmacology and Clinical Pharmacology, Pharmacogenomics Research Center, Inje University College of Medicine, Busan, Republic of Korea

**Keywords:** Tuberculosis, Non-tuberculosis mycobacterium, N-Acetyltransferase-2, Solute carrier organic anion transporter family member 1B1, The Center for Personalized Precision Medicine of Tuberculosis

## Abstract

**Background:**

The Center for Personalized Precision Medicine of Tuberculosis (cPMTb) was constructed to develop personalized pharmacotherapeutic systems for tuberculosis (TB). This study aimed to introduce the cPMTb cohort and compare the distinct characteristics of patients with TB, non-tuberculosis mycobacterium (NTM) infection, or latent TB infection (LTBI). We also determined the prevalence and specific traits of polymorphisms in N-acetyltransferase-2 (NAT2) and solute carrier organic anion transporter family member 1B1 (SLCO1B1) phenotypes using this prospective multinational cohort.

**Methods:**

Until August 2021, 964, 167, and 95 patients with TB, NTM infection, and LTBI, respectively, were included. Clinical, laboratory, and radiographic data were collected. NAT2 and SLCO1B1 phenotypes were classified by genomic DNA analysis.

**Results:**

Patients with TB were older, had lower body mass index (BMI), higher diabetes rate, and higher male proportion than patients with LTBI. Patients with NTM infection were older, had lower BMI, lower diabetes rate, higher previous TB history, and higher female proportion than patients with TB. Patients with TB had the lowest albumin levels, and the prevalence of the rapid, intermediate, and slow/ultra-slow acetylator phenotypes were 39.2%, 48.1%, and 12.7%, respectively. The prevalence of rapid, intermediate, and slow/ultra-slow acetylator phenotypes were 42.0%, 44.6%, and 13.3% for NTM infection, and 42.5%, 48.3%, and 9.1% for LTBI, respectively, which did not differ significantly from TB. The prevalence of the normal, intermediate, and lower transporter SLCO1B1 phenotypes in TB, NTM, and LTBI did not differ significantly; 74.9%, 22.7%, and 2.4% in TB; 72.0%, 26.1%, and 1.9% in NTM; and 80.7%, 19.3%, and 0% in LTBI, respectively.

**Conclusions:**

Understanding disease characteristics and identifying pharmacokinetic traits are fundamental steps in optimizing treatment. Further longitudinal data are required for personalized precision medicine.

**Trial registration:**

This study registered ClinicalTrials.gov NO. NCT05280886.

**Supplementary Information:**

The online version contains supplementary material available at 10.1186/s12890-023-02646-7.

## Background

Despite strenuous efforts, tuberculosis (TB) remains a significant public health concern. TB infection is highly contagious and should be diagnosed early and isolated to protect the surrounding community. Annually, 10 million cases of active TB and 1.3 million deaths are reported [1]. In 2022, there were 16,264 newly diagnosed TB patients, or 31.7 patients per 100,000 people, and 1,430 deaths were reported in South Korea [2]. The mainstay of the current standard TB treatment regimen is a four-drug combination consisting of isoniazid (INH), rifampicin (RIF), ethambutol (EMB), and pyrazinamide (PZA), which is highly effective. However, a considerable rate of treatment failure still occurs owing to adverse reactions of medication; consequently, many efforts have been made to optimize treatment regimens, including appropriate dosing strategies [3–6]. INH and RIF exhibit a high degree of pharmacokinetic variability [7–10] due to several factors, including drug formulation, age, sex, weight, treatment adherence, and comorbidities [9–12]. Moreover, pharmacogenetic variability in genes and encoding proteins for the metabolism and transportation of drugs, also contributes to this uncertainty [13, 14]. INH is a key drug in the standard regimen with bactericidal activity that rapidly reduces bacillary load. It is primarily metabolized by N-acetyltransferase type 2 (NAT2), which demonstrates substantial inter-individual variability in acetylating activities by *NAT2* genetic polymorphism [15]. As INH antimicrobial activity is correlated with drug concentration, suboptimal INH exposure may lead to treatment failure and emergence of drug resistance [5, 10, 16–18]. RIF is a critical drug for early sterilization of *Mycobacterium tuberculosi*s. RIF is metabolized by hepatic esterases and excreted by the biliary system. Organic anion transporting polypeptide 1B1 (OATP1B1*)* is a major membrane influx transporter that controls substrate uptake from the bloodstream into hepatocytes. The OATP1B1 transporter protein is encoded by solute carrier organic anion transporter family member 1B1 *(SLCO1B1)* gene, and reduced *SLCO1B1* expression and activity decreases RIF uptake, resulting in an increase in plasma RIF concentration.

The Center for Personalized Precision Medicine of Tuberculosis (cPMTb) cohort was constructed to develop personalized pharmacotherapy systems for TB. cPMTb cohort includes smaller numbers of patients with non-tuberculosis mycobacterium (NTM) infection and latent TB infection (LTBI). This study aimed to introduce the cPMTb cohort and describe the characteristic features of patients with TB compared with those with NTM infection or LTBI. Detailed characterization of these diseases may help to detect infectious diseases early, which would be critical to public health, as well as understanding the pathophysiology of each disease. Additionally, we identified the prevalence and characteristic traits of NAT2 and SLCO1B1 polymorphic phenotypes.

## Methods

### Study design and the cPMTb Cohort database

The cPMTb workstation is a centralized, interactive, and multifunctional R&D system designed to enhance TB management. The detailed cPMTb study design has been described previously [19]. Briefly, cPMTb is a multinational prospective cohort comprising adult TB patients aged > 15 years. A website (https://smart.cpmtb.kr/#/cohort/status) provides a real-time summary of cohort data, and authorized users have access to additional information or interactive functions. Participants from the cPMTb cohort were included in the current analysis (data freeze, August 10, 2021). According to Korean TB guidelines [20], all patients with TB were monitored at regular intervals during their anti-TB treatment. Baseline characteristics, including age, sex, body mass index (BMI), and comorbidities were recorded. History of anti-TB treatment and sites of TB involvement were also recorded. In addition, the results of laboratory, microbiological, and radiographic tests, including the presence of cavities, were obtained prior to treatment initiation. This study adheres to the principles of the Declaration of Helsinki. The Institutional Review Board of all participating sites reviewed and approved the study protocol. All the participants provided written informed consent.

### Diagnostic criteria for TB, NTM, and LTBI

TB was diagnosed by isolating *Mycobacterium tuberculosis* from sputum, body fluid, or tissue biopsy. Suspected pulmonary TB patients may undergo acid-fast bacilli (AFB) smear and culture tests to confirm the presence of isolated mycobacterium. If molecular tests, including the nucleic acid amplification test or tissue biopsy reveal characteristic findings, such as granulomatous inflammation accompanied by caseous necrosis, TB could be diagnosed [21, 22].

The diagnosis of NTM must be distinguished from contamination and colonization of sputum cultures. It can be diagnosed if one or more sputum cultures through bronchoscopy or repeated sputum cultures identify the same NTM. In the case of NTM lung disease, initiation of treatment was clinically decided when respiratory symptoms and radiologic characteristics worsened, including cavitary and bronchiectasis lesions [23, 24].

For diagnosing LTBI, excluding active TB infection is the most important criteria. The tests are conducted on high-risk subjects, such as those in close contact with active TB and those with immunodeficiency. To diagnose LTBI, one of the tuberculin skin test (TST) or Interferon-gamma release assay (IGRA) tests was performed [25, 26].

### Determination of pharmacokinetic genotypes and phenotypes

Genomic DNA was extracted from whole blood using the Blood Genomic DNA Miniprep Kit (Cosmo genetech, Seoul, Republic of Korea) according to the manufacturer’s instructions. Single nucleotide polymorphisms (SNPs) in *NAT2* and *SLCO1B1* variants were assessed using the SNaPshot Multiplex Kit (Applied Biosystems, Foster City, CA). *NAT2* genetic polymorphisms were analyzed at the six most common SNP sites (rs1801279 for 191G > A, rs1041983 for 282C > T, rs1801280 341 T > C, rs1799930 for 590G > A, rs1208 for 803A > G, and rs1799931 for 857G > A) and categorized as rapid, intermediate, and slow/ultra-slow acetylator phenotypes, resulting in a trimodal distribution of INH elimination [27, 28]. Slow/ultra-slow acetylators have a higher risk of adverse drug reactions; however, rapid acetylators may encounter diminished clinical efficacy such as treatment failure [27–31]. The phenotypes of *NAT2* acetylators were classified using NAT2PRED (http://nat2pred.rit.albany.edu/) [19]. *SLCO1B1* genetic polymorphism was evaluated at two SNP sites (ex. rs2306283 for 388A > G, rs4149056 for 521 T > C) and categorized into normal, intermediate, and low transporter functions.

### Statistical analysis

Patient characteristics are presented as mean (± standard deviation) or median (interquartile ranges) for continuous variables, and relative frequencies for categorical variables. Continuous variables were compared using the *t*-test or Wilcox rank-sum test, and categorical variables using the chi-squared test or Fisher’s exact test. A correlation network was constructed by Pearson’s correlation using the igraph package. Each item was represented by a node, whose size indicated the prevalence. The links between the nodes indicated statistically significant correlation, while the thickness represented the strength of the correlation. The variables were chosen based on the least absolute shrinkage and selection operation (LASSO) regression analysis using the glmnet package. Logistic regression was performed for multivariable analysis. To compare the discrimination power of each model, the area under the curve (AUC) of the receiver operating characteristic (ROC) curve was calculated using the ROCR package. To assess predictive accuracy, fivefold cross validation was performed using the boot package. The Brier score was used to determine the model’s calibration. When the Brier score was less than 0.25, the model was considered to be calibrated properly. All statistical analyses were performed using R software (version 3.6.0).

## Results

### Baseline characteristics of enrolled patients

Of the 1696 patients registered as of August 10, 2021, 1226 Korean participants from 19 respiratory centers of university hospitals in the Republic of Korea were included for this study. Among them, 964, 167, and 95 patients had TB, NTM infection, and LTBI, respectively (Supplementary Figure S1). In addition, 43 Chinese and 222 Southeast Asian TB patients were also included in the database but were excluded from the final analysis due to the absence of comparison subjects. The demographic, laboratory, and radiographic characteristics of these patients are described in Table 1. In patients with TB, the median age was 58 years; these patients were older than patients with LTBI but younger than patients with NTM infection. The proportion of male patients was higher in the TB group (67.2%) than in the NTM (44.3%) or LTBI (43.2%) groups. The median BMI of the TB group was 21.5 kg/m^2^, which was higher than that of the NTM group but lower than that of the LTBI group. Diabetes was the most prevalent comorbidity (26.0%) among the groups. Regarding the site of involvement in TB patients, 854 (88.6%) patients had lung involvement, 52 (5.4%) had pleural effusion, 6 (0.6%) had endobronchial TB, 21 (2.2%) had TB lymphadenitis, 7 (0.7%) had miliary TB, 7 (0.7%) had abdominal TB, 1 (0.1%) had bone/joint TB, and 1 (0.1%) had brain involvement. In the NTM group, 32 (19.2%) patients had *M.avium* infection, 58 (34.7%) had *M.intracellulare*, 3 (1.7%) had *M.abscessus*, 6 (3.6%) had *M.massiliense*, and 8 (4.8%) had *M.kansassi*.

**Table 1:** Demographic characteristics of total study population

	**Total**	**TB**	**NTM**	**LTBI**	**P-value**		
	(*N* = 1226)	(*N *= 964)	(*N* = 167)	(*N* = 95)	TB vs. NTM	TB vs. LTBI	NTM vs. LTBI
**Demographics**							
Age	58 [48, 70]	60 [47, 70]	65 [57, 73]	53 [42.5, 60]	< 0.001	< 0.001	< 0.001
Male sex	848 (61.8%)	646 (67.2%)	74 (44.3%)	41 (43.2%)	< 0.001	< 0.001	0.959
Body mass index, kg/m^2^	21.5 [19.3, 23.6]	21.5 [19.4, 23.8]	20.0 [18.6, 22.3]	23.0 [20.4, 24.8]	< 0.001	< 0.001	< 0.001
**Comorbidity**							
Previous TB history	213 (15.8%)	149 (15.6%)	42 (25.9%)	0 (0.0%)	0.002	< 0.001	< 0.001
Diabetes	173 (21.7%)	147 (26.0%)	8 (6.6%)	5 (11.4%)	< 0.001	0.047	0.500
COPD	23 (2.9%)	12 (2.1%)	7 (5.8%)	1 (2.3%)	0.055	> 0.999	0.604
Hypertension	155 (19.4%)	110 (19.5%)	26 (21.1%)	6 (13.6%)	0.704	0.453	0.365
**Laboratory findings**							
WBC, /μL×1000	6.2 [5.0, 7.9]	6.4 [5.1, 8.1]	6.1 [5.0, 7.6]	5.7 [4.7, 6.9]	0.185	0.003	0.098
Neutrophil, %	62.0 [53.7, 69.8]	62.5 [53.9, 70.5]	61.7 [54.3, 69.0]	56.7 [51.2, 62.9]	0.535	< 0.001	0.005
Lymphocyte, %	25.7 ± 10.7	25.1 ± 11.0	26.7 ± 10.1	31.3 ± 8.1	0.098	< 0.001	0.001
Hb, g/dL	13.1 ± 1.8	13.0 ± 1.9	12.9 ± 1.6	14.0 ± 1.4	0.732	< 0.001	< 0.001
Platelet, /μL×1000	265.8 ± 97.6	268.8 ± 98.7	252.2 ± 97.3	232.2 ± 57.1	0.049	< 0.001	0.041
Albumin, g/dL	4.2 [3.8, 4.4]	4.1 [3.7, 4.4]	4.2 [3.9, 4.4]	4.4 [4.2, 4.5]	0.035	< 0.001	< 0.001
Protein, g/dL	7.2 [6.8, 7.6]	7.2 [6.8, 7.7]	7.3 [6.95, 7.7]	7.2 [7.0, 7.45]	< 0.001	0.100	0.047
**Radiographic feature**							
Cavity	114 (8.3%)	84 (8.7%)	20 (12.0%)	0 (0.0%)	0.229	0.005	0.001

Abbreviations: *TB* Tuberculosis, *NTM* Non-tuberculosis mycobacterium, *LTBI* Latent tuberculosis infection, *WBC* White blood cell count, *Hb* Hemoglobin

**Fig. 1:**
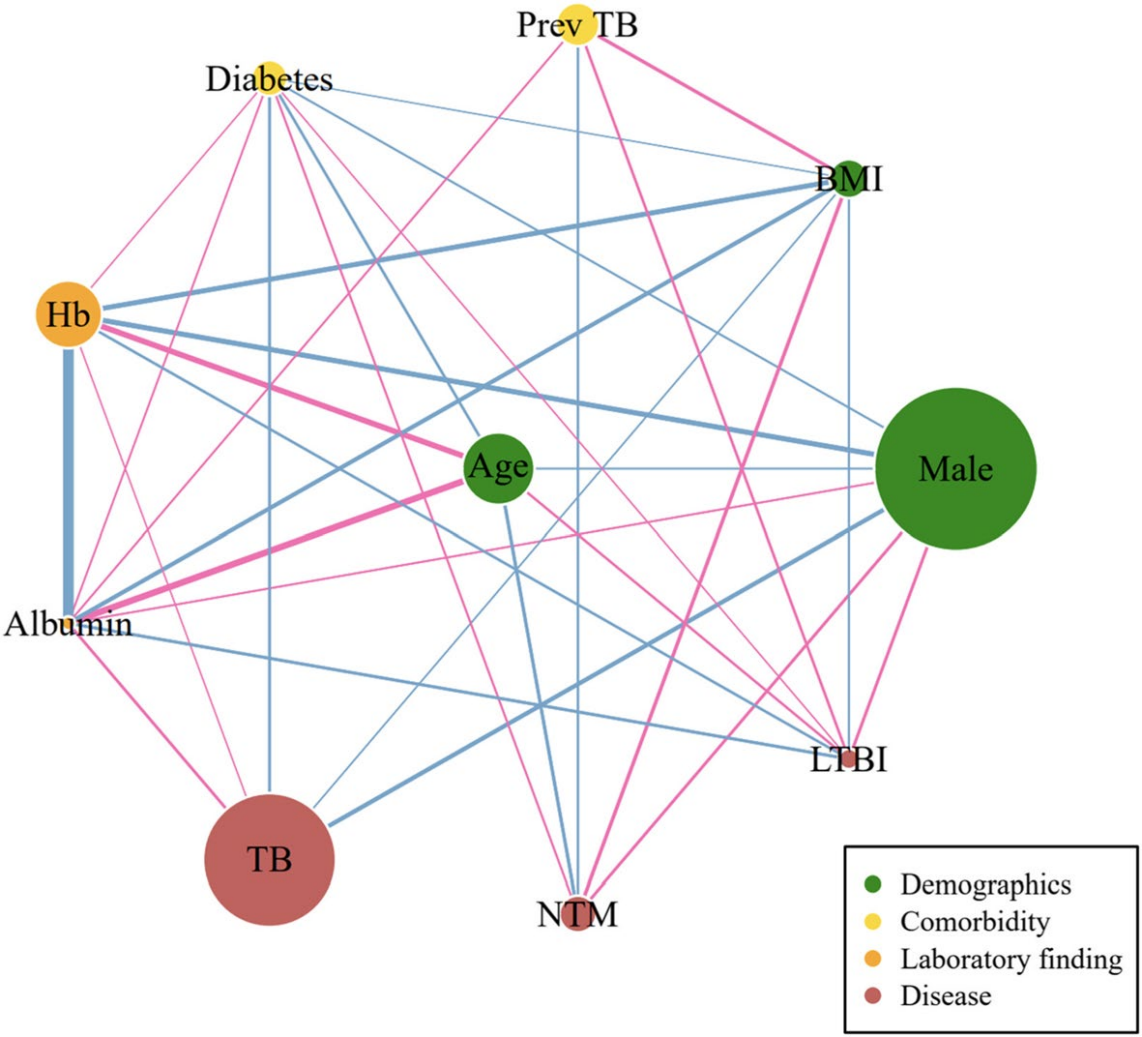
Network analysis

The median age of the NTM group was 65 years, which was the oldest group in our cohort, and 93 patients (55.7%) were female. The median BMI was 20.0 kg/m^2^, which was the lowest value, and 42 (25.9%) had a previous history of TB, which was the highest frequency among the groups. Hypertension was the most prevalent comorbidity in the NTM group (21.1%), although the prevalence of COPD was highest among all the groups. In contrast to patients with TB, the prevalence of diabetes was low in patients with NTM (6.6%). The age group distribution of TB, NTM infection, and LTBI, stratified by sex, is shown in Supplementary Figure S2. The lowest albumin levels and highest platelet counts were found in patients with TB, followed by those with NTM infection and LTBI. Patients with TB and NTM infection had higher white blood cell count (WBC) and neutrophil percentage and a lower lymphocyte percentage and hemoglobin (Hb) levels than patients with LTBI. Regarding radiographic features, 85 (8.7%) and 20 (12.0%) patients with TB and NTM infection, respectively, had cavitary lesions, which were not statistically different. Correlation network demonstrating the inter-relationship between variables is shown in Fig. 1.

### Multivariable analysis

Using the LASSO regression analysis, younger age, higher BMI, no previous TB history, the presence of diabetes, and lower albumin level were significantly associated with TB disease versus NTM infection for prediction model. The AUC of the ROC curve for this model was 0.773, and the predictive accuracy was 0.849. Male sex, current smoking status, presence of diabetes, and lower hemoglobin level were significantly associated with patients with TB versus LTBI patients. The AUC for this model was 0.703, and the predictive accuracy was 0.892. Patients with NTM were older, had lower Hb level, and a higher platelet level than patients with LTBI. The AUC for this model was 0.883, and the predictive accuracy was 0.834. The procedures for selecting variables are shown in Supplemental Figure S3. The results of the logistic regression analysis and the ROC curve are summarized in Table 2 and Supplemental Figure S4, respectively. The Brier scores of models for TB vs. NTM, TB vs. LTBI, and NTM vs. LTBI were 0.136, 0.091, and 0.139, which demonstrated their good calibration.

**Table 2:** Multivariable analysis for each model

	**OR**	**95% CI**
**TB vs. NTM**		
Age	0.944	0.911–0.978
Body mass index	1.154	1.034–1.289
Previous TB history	0.407	0.162–1.022
Diabetes	4.062	1.236–13.348
Albumin	0.417	0.191–0.911
**TB vs. LTBI**		
Male sex	1.918	0.579–6.355
Current smoker	2.652	1.107–6.349
Diabetes	3.268	0.716–1.491
Hemoglobin	0.392	0.237–0.650
**NTM vs. LTBI**		
Age	1.108	1.036–1.186
Hemoglobin	0.474	0.265–0.847
Platelet	1.013	1.000–1.026

Abbreviations: *TB* Tuberculosis, *NTM* Non-tuberculosis mycobacterium, *LTBI* Latent tuberculosis infection, *OR* Odds ratio, *CI* Confidential interval, *AST* Aspartate aminotransferase

### Subgroups according to NAT2 and SLCO1B1 phenotypes

**Fig. 2:**
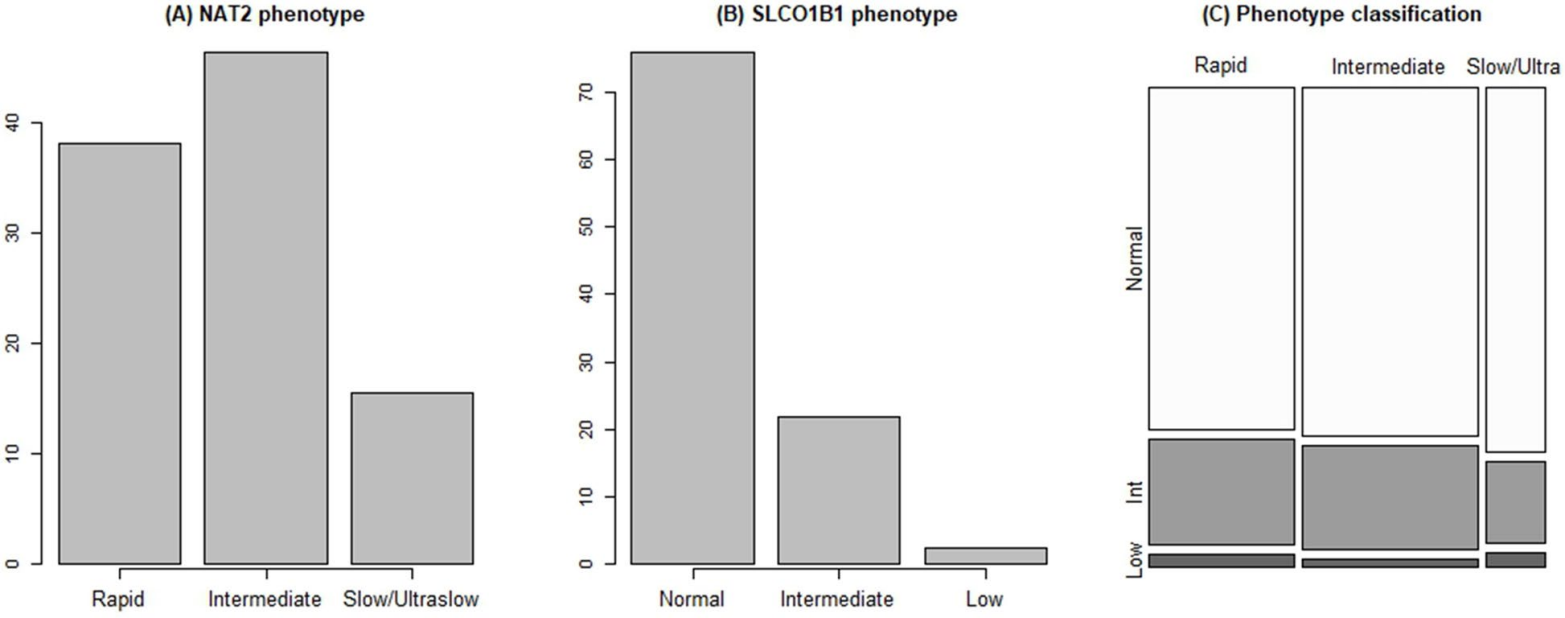
Frequencies of (**A**) *NAT2* phenotype, (**B**) *SLCO1B1* phenotype, and (**C**) their cross table in patients with tuberculosis. Abbreviations: *NAT*2 N-acetyltransferase type 2, *SLCO1B1* Solute carrier organic anion transporter family member 1B1

Of the 964 patients with TB, 921 were successfully assessed for the *NAT2* genotype. Of these, 361 (39.2%), 443 (48.1%), and 117 (12.7%) patients were rapid, intermediate, and slow/ultra-slow acetylators, respectively (Fig. 2A). In addition, 919 patients were assessed for the *SLCO1B1* genotype: 688 (74.8%), 209 (22.7%), and 22 (2.4%) patients had normal, intermediate, and low transporter functions, respectively (Fig. 2B). The cross-table for the frequencies of both the NAT2 and SLCO1B1 phenotypes is summarized in Fig. 2C. The prevalence of rapid, intermediate, and slow/ultra-slow acetylator phenotypes were 42.0%, 44.6%, and 13.3% for NTM infection, and 42.5%, 48.3%, and 9.1% for LTBI, respectively, which did not differ significantly from TB. The prevalence of the normal, intermediate, and lower transporter SLCO1B1 phenotypes in TB, NTM, and LTBI did not differ significantly; 74.9%, 22.7%, and 2.4% in TB; 72.0%, 26.1%, and 1.9% in NTM; and 80.7%, 19.3%, and 0% in LTBI, respectively. The SNP genotypes and phenotypes are detailed in Supplemental Table S1. The baseline characteristics, including SNP phenotypes, of Korean, Chinese, and Southeast Asian TB patients are compared in Supplemental Table S2. The prevalence of slow/ultraslow acetylator was higher in Southeast Asian patients compared to Korean or Chinese patients. On the contrary, the prevalence of the normal SLCO1B1 phenotype was greater among Southeast Asian patients than among Korean or Chinese patients.

The baseline characteristics of patients with TB according to the NAT2 phenotypes are summarized in Table 3. No differences were observed between the three groups in demographic characteristics, including age, sex, smoking status, BMI, and comorbidities; however, hypertension prevalence was higher in the rapid acetylator group (22.0%) than in the intermediate acetylator group (12.3%), despite a similar age distribution. For the drug adverse events, higher any adverse event was reported in slow/ultra-slow acetylator group (25.6%) compared to rapid acetylator group (16.6%; *P* = 0.042). Among them, hepatotoxicity was significantly higher in intermediate (5.4%) and slow/ultra-slow acetylators (16.2%) compared to rapid acetylators (4.4%). Additionally, skin rash was higher in intermediate acetylator group (5.4%) compared to rapid acetylator group (2.2%; *P* = 0.033). The baseline characteristics of patients with TB according to the SLCO1B1 phenotypes are summarized in Supplemental Table S3. None of these variables including adverse events differed significantly between the SLCO1B1 phenotype groups. The AUC of the ROC curve for the NAT2 and SLCO1B1 phenotypes to predict hepatotoxicity was 0.619 and 0.528, respectively (Supplemental Figure S5).

**Table 3:** Baseline characteristics and adverse events of tuberculosis patients according to NAT2 phenotype

	***NAT2 phenotype***			***P-values***		
	**Rapid acetylator** (*N* = 361)	**Intermediate** (*N* = 443)	**Slow/ultraslow** (*N* = 117)	*Rapid vs. intermediate*	*Rapid vs. slow*	*Intermediate vs. slow*
**Demographics**						
Age	59 [47,69]	60 [47,72]	60 [46,68]	0.664	0.989	0.824
Male sex	247 (68.4%)	293 (66.1%)	79 (67.5%)	0.527	0.946	0.852
Body mass index (kg/m^2^)	21.3 [19.2, 23.5]	21.8 [19.4, 23.8]	21.8 [19.6, 23.7]	0.090	0.286	0.987
Previous TB history	63 (17.5%)	62 (14.2%)	19 (16.2%)	0.232	0.863	0.675
**Comorbidity**						
Diabetes	52 (24.5%)	70 (27.9%)	16 (21.9%)	0.477	0.770	0.386
COPD	2 (0.9%)	9 (3.6%)	1 (1.4%)	0.120	> 0.999	0.563
HTN	50 (23.6%)	36 (14.3%)	15 (20.5%)	0.015	0.710	0.272
**Laboratory findings**						
WBC, /μL*1000	6.2 [5.0, 7.8]	6.5 [5.17, 8.16]	6.3 [5.1, 8.4]	0.271	0.728	0.738
Hb, g/dL	13.0 ± 1.9	13.0 ± 1.9	13.0 ± 2.0	0.524	0.812	0.856
Platelet, /μL*1000	262 ± 99	274 ± 96	268 ± 99	0.101	0.547	0.624
Albumin, g/dL	4.1 [3.6, 4.4]	4.1 [3.8, 4.4]	4.1 [3.7, 4.4]	0.456	0.984	0.672
Protein, g/dL	7.2 [6.8, 7.6]	7.2 [6.9, 7.7]	7.1 [6.8, 7.7]	0.150	0.906	0.254
AST, U/L	25 [19, 31]	25 [19, 32]	24 [20, 37]	0.323	0.156	0.412
ALT, U/L	18 [12, 26.5]	18 [13, 26]	18 [13, 34]	0.756	0.195	0.268
**Radiographic**						
Cavity	30 (8.3%)	43 (9.7%)	9 (7.7%)	0.574	0.986	0.625
**Adverse events**						
Any	60 (16.6%)	86 (19.4%)	30 (25.6%)	0.353	0.042	0.177
Hepatotoxicity	16 (4.4%)	24 (5.4%)	19 (16.2%)	0.634	< 0.001	< 0.001
Skin rash	8 (2.2%)	24 (5.4%)	4 (3.4%)	0.033	0.702	0.520
GI trouble	18 (5.0%)	29 (6.5%)	11 (9.4%)	0.431	0.130	0.387
CBC abnormalities	6 (1.7%)	6 (1.4%)	1 (0.9%)	0.948	0.850	> 0.999
Fever	0 (0%)	4 (0.9%)	1 (0.9%)	0.192	0.552	> 0.999
Arthralgia	8 (2.2%)	8 (1.8%)	1 (0.9%)	0.873	0.582	0.753
Neuropathy	6 (1.7%)	1 (0.2%)	1 (0.9%)	0.072	0.850	0.886

Abbreviations: *TB* Tuberculosis, *NTM* Non-tuberculosis mycobacterium, *LTBI* Latent tuberculosis infection, *BMI* Body mass index, *COPD* Chronic obstructive pulmonary disease, *HTN* Hypertension, *BPH* Benign prostate hyperplasia, *WBC* White blood cell count, *Hb* Hemoglobin, *BUN* Blood urea nitrogen, *Cr* Creatinine, *AST* Aspartate aminotransferase, *ALT* Alkaline aminotransferase, *CBC* Complete blood count

## Discussion

This study aimed to introduce the cPMTb cohort database and compare the clinical characteristics of patients with TB, NTM infection, and LTBI to determine their differential features. Patients with TB were younger and had higher male proportion and diabetes prevalence than patients with NTM infection. Patients with NTM infection had the lowest BMI, although patients with TB had the lowest albumin levels and highest platelet counts. WBC count and neutrophil percentage increased, while lymphocyte percentage and Hb level decreased in patients with TB and NTM infection. Furthermore, NAT2 polymorphism prevalence in Korea was 39.2%, 48.1%, and 12.7% for rapid, intermediate, and slow/ultra-slow acetylator phenotypes, respectively. SLCO1B1 polymorphism prevalence was 74.9%, 22.7%, and 2.4% for normal, intermediate, and low transporter function, respectively.

Numerous studies have explained active TB progression in terms of host-environment interactions when the patient is exposed to *M. tuberculosis* via droplets or aerosols; primary infection occurs according to host CD4 T lymphocyte and macrophage immune responses [32]. In addition, host factors such as HIV infection [33], low BMI [34], malnutrition [32], and comorbidities [35] can contribute to LTBI progression to active TB infection. The association between low BMI and host susceptibility to active TB is well-known [36], however, in our study, patients with NTM infection had a lower BMI than patients with TB. Diabetes has often been cited as a risk factor for TB [37, 38], however, our study suggested a differential impact on NTM disease. Since BMI and diabetes are related to metabolic syndrome [39], investigating the complex relationship between BMI, diabetes, TB, and NTM disease is necessary. In our cPMTb cohort, patients with TB presented with hypoalbuminemia, reflecting poor nutritional status, even when compared with patients with NTM infection and low BMI. Low albumin levels increase in-hospital mortality in patients with TB [40, 41] and negatively impact the treatment process and poor prognosis [42].

In our study, the slow/ultra-slow NAT2 phenotype prevalence was 12.7% in Koreans, and *NAT2* genotype distribution did not differ between the TB, NTM, and LTBI groups. The *NAT2* genotype is an autosomal recessive trait that varies by race and ethnicity. A recent systemic review showed that East Asians have the highest frequency of the fast acetylator phenotype, but no comparisons between countries were made [43]. Detecting NAT2 phenotypes has been considered from the beginning of tuberculosis treatment in order to avoid drug-related liver injury and determine appropriate dosing [44, 45]. The *NAT2* slow acetylator prevalence is estimated to be 40–70% in Caucasians and less prevalent in Asians [46]. The frequency of the slow *NAT2* genotype in the Chinese population was 25.4% [47]. The frequency of the *NAT2* slow acetylator group was reported to be 6.8% in Japan and 22.4% in Thailand in a study with a small sample size [48, 49]. The NAT2 enzyme, encoded by the *NAT2* gene in the liver, is involved in the metabolism and detoxification of carcinogenic arylamines and drugs. The *NAT2* slow acetylator phenotype is associated with cancer risk and adverse drug reactions. An early study from the United States estimated that *NAT2* slow acetylator distribution in Korean ancestry was approximately 32%, and liver injury risk was higher in the slow acetylator group [50]. In contrast, the NAT2 slow acetylator group comprised 14.4% of the total in a Korean study on anti-TB drug-related hepatotoxicity [51]. In our large nationwide cohort, NAT2 slow/ultra-slow acetylators represented 12.5% of the total population. We also examined the *SLCO1B1* genotype polymorphism, which is believed to influence drug-related side effects similar to those of the NAT2 phenotype [52, 53]. Despite insufficient evidence of clinical efficacy, initial and adjustment dosing models may be required to reduce the adverse effects of RIF [54]. Previously, there have been studies on SLCO1B1 variants in Thai and distinct Asian populations. However, few studies have yet related these phenotypes to clinical conditions [55, 56].

The greatest strength of our study is that we determined the prevalence and characteristics of *NAT2* and *SLCO1B1* phenotypes in a large population through a nationwide cohort study. Previous studies have investigated patients’ genetic phenotypes of NAT2 and SLCO1B1 with small numbers of samples [54, 57], and compared to previous reports, our cohort includes one of the largest numbers of patients who have undergone genetic testing. There had been few domestic studies on anti-TB drugs and genotypes [58], despite having the highest TB prevalence and mortality among Organization for Economic Co-operation and Development (OECD) countries. Consequently, this study may serve as a cornerstone for personalized precision medicine. In contrast to the other TB cohorts, the cPMTb cohort included patients with NTM infection and LTBI, allowing for direct comparison of their characteristics. Furthermore, we developed prediction models for TB and NTM with high accuracy and validity for early diagnosis even before receiving an AFB culture report. Additionally, clinical characteristics, such as demographics, nutrition status, and comorbidities, were identified to understand the pathophysiology of disease development. A better understanding of this mechanism may provide further solutions for TB management.

However, this study had some limitations. First, association between plasma drug concentrations and serial follow-up data was not reflected. Second, we included patients with TB, NTM, and LTBI in our cohort and described their disease burden. Due to the lack of national prevalence data for NTM or LTBI, we were unable to compare the prevalence of NTM and LTBI in our cohort. Moreover, there is a gap between the time of diagnosis and treatment initiation for patients with NTM and LTBI, and it is difficult to define the overall disease burden due to the nature of the disease. Third, because we did not collect detailed radiographic characteristics, such as the number or diameter of cavities or the extent of disease, analyses of such characteristics were limited. As adverse effects increase with treatment duration, additional research should be conducted on these *NAT2* and *SLCO1B1* genotypes with prolonged treatment. Finally, the collection of clinical outcome data is still in progress, so inferring a clinical prognosis is limited. Therefore, long-term monitoring of our cohort is necessary.

## Conclusions

In conclusion, patients with Tb were older, had lower BMI and higher male proportion and diabetes than patients with LTBI. However, patients with NTM were older, had lower BMI, male proportion, and diabetes rate, and higher previous TB history compared to patients with TB. Patients with TB had the lowest albumin levels, and the prevalence of the rapid, intermediate, and slow/ultra-slow acetylator phenotypes were 39.2%, 48.1%, and 12.7%, respectively. Understanding disease characteristics and identifying the pharmacokinetics are fundamental to optimizing treatment. Further research on acetylator-specific dose adjustments based on pharmacokinetic phenotypes is required for personalized precision medicine in TB.

### Supplementary Information

**Additional file 1. **

## Data Availability

Due to its proprietary nature, supporting data cannot be made openly available. Further information about the data and conditions for access are available at the pCMPb (https://www.cpmtb.kr).

## Abbreviations

cPMTb: Center for Personalized Precision Medicine of Tuberculosis
TB: Tuberculosis
NTM: Non-tuberculosis mycobacterium
LTBI: Latent TB infection
NAT2: N-acetyltransferase-2
SLCO1B1: Solute carrier organic anion transporter family member 1B1
BMI: Body mass index
INH: Isoniazid
RIF: Rifampicin
EMB: Ethambutol
PZA: Pyrazinamide
SNP: Single nucleotide polymorphism
AFB: Acid-fast bacilli
AUC: Area under the curve
ROC: Receiver operating characteristic
HIV: Human immunodeficiency virus
