# Supplementary material for: Risk adjustment model for tuberculosis compared to non-tuberculosis mycobacterium or latent tuberculosis infection: Center for Personalized Precision Medicine of Tuberculosis (cPMTb) cohort database
Source: BMC Pulm Med. 2023 Nov 24;23:471. doi: 10.1186/s12890-023-02646-7 (PMC10675857; doi:10.1186/s12890-023-02646-7)

**SUPPLEMENTS**

**Supplementary Table S1**. Genotype and phenotype characteristics of total study population

|  |  | **Total** | **TB** | **NTM** | **LTBI** | *P-value* |
| --- | --- | --- | --- | --- | --- | --- |
|  |  | (N=1485) | (N=1226) | (N=165) | (N=94) |  |
| *NAT2 genotype* | |  |  |  |  | 0.844 |
| 1 | *4(wt)/*4(wt) | 530 (40.7%) | 356 (38.7%) | 68 (43.3%) | 39 (44.3%) |  |
| 2 | *4(wt)/*12 | 4 (0.3%) | 3 (0.3%) | 0 (0.0%) | 0 (0.0%) |  |
| 3 | *4(wt)/*13 | 4 (0.3%) | 3 (0.3%) | 0 (0.0%) | 0 (0.0%) |  |
| 5 | *4(wt)/*5 | 31 (2.4%) | 26 (2.8%) | 1 (0.6%) | 1 (1.1%) |  |
| 6 | *4(wt)/*6 | 335 (25.7%) | 247 (26.8%) | 39 (24.8%) | 23 (26.1%) |  |
| 7 | *4(wt)/*7 | 234 (18.0%) | 168 (18.2%) | 29 (18.5%) | 17 (19.3%) |  |
| 8 | *6/*12 | 1 (0.1%) | 1 (0.1%) | 0 (0.0%) | 0 (0.0%) |  |
| 10 | *5/*6 | 14 (1.1%) | 11 (1.2%) | 1 (0.6%) | 1 (1.1%) |  |
| 11 | *5/*7 | 8 (0.6%) | 4 (0.4%) | 3 (1.9%) | 0 (0.0%) |  |
| 12 | *6/*6 | 54 (4.2%) | 35 (3.8%) | 6 (3.8%) | 4 (4.5%) |  |
| 13 | *6/*7 | 67 (5.1%) | 51 (6.5%) | 9 (5.7%) | 2 (2.2%) |  |
| 14 | *7/*7 | 19 (1.5%) | 16 (1.7%) | 1 (0.6%) | 1 (1.1%) |  |
| NAT2 phenotype | |  |  |  |  | 0.479 |
|  | Rapid acetylator | 535 (41.1%) | 361 (39.2%) | 66 (42.0%) | 37 (42.5%) |  |
|  | Intermediate | 604 (46.4%) | 443 (48.1%) | 70 (44.6%) | 42 (48.3%) |  |
|  | Slow/ultraslow | 162 (12.5%) | 117 (12.7%) | 21 (13.3%) | 8 (9.1%) |  |
| *SLCO1B1 genotype* | |  |  |  |  | 0.170 |
| 1 | *1a/*1a | 82 (6.3%) | 55 (6.0%) | 14 (8.9%) | 4 (4.5%) |  |
| 2 | *1a/*1b | 397 (30.6%) | 270 (29.4%) | 49 (31.2%) | 35 (39.8%) |  |
| 3 | *1a/*5 | 1 (0.1%) | 1 (0.1%) | 0 (0.0%) | 0 (0.0%) |  |
| 4 | *1a/15 | 108 (8.3%) | 73 (8.0%) | 15 (9.6%) | 11 (12.5%) |  |
| 5 | *1b/*1b | 487 (37.7%) | 361 (39.3%) | 49 (31.2%) | 32 (36.4%) |  |
| 6 | *1b/*15 | 192 (14.8%) | 137 (14.9%) | 27 (17.2%) | 6 (6.8%) |  |
| 8 | *15/*15 | 29 (2.2%) | 21 (2.3%) | 3 (1.9%) | 0 (0.0%) |  |
| SLCO1B1 phenotype | |  |  |  |  | 0.410 |
|  | Normal | 971 (74.7%) | 688 (74.9%) | 113 (72.0%) | 71 (80.7%) |  |
|  | Intermediate | 298 (22.9%) | 209 (22.7%) | 41 (26.1%) | 17 (19.3%) |  |
|  | Low transporter | 30 (2.3%) | 22 (2.4%) | 3 (1.9%) | 0 (0.0%) |  |

Abbreviations: TB, tuberculosis; NTM, non-tuberculosis Mycobacterium; LTBI, latent tuberculosis infection; NAT2, N-acetyltransferase type 2; SLCO1B1, solute carrier organic anion transporter family member 1B1

**Supplemental Table S2**. Comparison of demographic characteristics, including SNP phenotypes, across ethnic groups

|  | Total | Korea | Chinese | Southeast Asia | P-value | | |
| --- | --- | --- | --- | --- | --- | --- | --- |
|  | (N=1229) | (N=964) | (N=43) | (N=222) | Korea vs. Chinese | Korean vs. Southeast | Chinese vs.  Southeast |
| Demographics |  |  |  |  |  |  |  |
| Age | 56 [43, 67] | 60 [47, 70] | 53 [40, 63] | 45 [40.3, 54.75] | 0.01 | <0.001 | 0.002 |
| Male sex | 805 (65.6%) | 646 (67.2%) | 28 (65.1%) | 131 (59.0%) | 0.911 | 0.026 | 0.563 |
| BMI, kg/m^2^ | 21.1 [18.8, 23.4] | 21.5 [19.4, 23.8] | 22 [19.7, 23.4] | 18.6 [16.8, 20.9] | 0.956 | <0.001 | <0.001 |
| Comorbidity |  |  |  |  |  |  |  |
| Previous TB | 229 (18.7%) | 149 (15.6%) | 10 (23.3%) | 69 (31.4%) | 0.255 | <0.001 | 0.379 |
| Diabetes | 215 (29.8%) | 147 (26.0%) | 2 (16.7%) | 66 (45.8%) | 0.690 | <0.001 | 0.098 |
| COPD | 15 (2.1%) | 12 (2.1%) | 2 (16.7%) | 1 (0.7%) | 0.022 | 0.428 | 0.005 |
| Hypertension | 117 (16.2%) | 110 (19.5%) | 4 (33.3%) | 3 (2.1%) | 0.408 | <0.001 | <0.001 |
| Lab findings |  |  |  |  |  |  |  |
| WBC, /μL*1000 | 6.6 [5.3, 8.5] | 6.4 [5.1, 8.1] | 6.3 [5.0, 7.2] | 8.0 [6.5, 11.1] | 0.173 | <0.001 | <0.001 |
| Neutrophil, % | 64 [54.9, 72.8] | 62.6 [53.9, 70.5] | 57 [51.4, 66.3] | 72 [58.9, 82.8] | 0.103 | <0.001 | <0.001 |
| Lymphocyte, % | 23.9 ± 11.4 | 25.1 ± 11.0 | 28.4 ± 10.7 | 18.0 ± 11.5 | 0.055 | <0.001 | <0.001 |
| Hb, g/dL | 12.8 ± 2.1 | 13.0 ± 1.9 | 13.8 ± 1.8 | 11.6 ± 2.3 | 0.008 | <0.001 | <0.001 |
| Platelet, /μL*1000 | 276.8 ± 105.3 | 268.8 ± 98.7 | 268.6 ± 75.0 | 313.4 ± 128.6 | 0.991 | <0.001 | 0.003 |
| Albumin, g/dL | 4.0 [3.5, 4.3] | 4.1 [3.7, 4.4] | 4.2 [4.0, 4.4] | 3.3 [2.9, 3.5] | 0.147 | <0.001 | <0.001 |
| Radiographic |  |  |  |  |  |  |  |
| Cavity | 168 (13.7%) | 84 (8.7%) | 3 (7.0%) | 81 (36.5%) | 0.905 | <0.001 | <0.001 |
| SNP Phenotype |  |  |  |  |  |  |  |
| NAT2 |  |  |  |  | 0.090 | <0.001 | <0.001 |
| Rapid | 414 (36.8%) | 361 (39.2%) | 23 (56.1%) | 30 (15.9%) |  |  |  |
| Intermediate | 547 (47.5%) | 443 (48.1%) | 15 (36.6%) | 89 (47.1%) |  |  |  |
| Slow/ultraslow | 190 (16.5%) | 117 (12.7%) | 3 (7.3%) | 70 (37.0%) |  |  |  |
| SLCO1B1 |  |  |  |  | 0.993 | 0.110 | 0.011 |
| Normal | 874 (76.1%) | 688 (74.9%) | 31 (75.6%) | 155 (82.0%) |  |  |  |
| Intermediate | 249 (21.7%) | 209 (22.7%) | 9 (22.0%) | 31 (16.4%) |  |  |  |
| Low | 26 (2.3%) | 22 (2.4%) | 1 (2.4%) | 3 (1.6%) |  |  |  |

**Supplementary Table S3**. Baseline characteristics of patients with tuberculosis according to the SLCO1B1 phenotype

|  |  | ***SLCO1B1 phenotype*** | |  |  |
| --- | --- | --- | --- | --- | --- |
|  |  | **Normal** | **Intermediate** | **Low** | *P-value* |
|  |  | (N=688) | (N=209) | (N=22) |  |
| **Demographics** | |  |  |  |  |
| Age |  | 59 [47.8, 70] | 60 [42, 70] | 59 [51, 70] | 0.560 |
| Sex | Male | 466 (67.8%) | 139 (66.5%) | 12 (54.5%) | 0.413 |
|  | Female | 221 (32.2%) | 70 (33.5%) | 10 (45.5%) |  |
| Smoking status | |  |  |  | 0.267 |
|  | Never | 307 (44.8%) | 96 (46.4%) | 12 (54.5%) |  |
|  | Ex | 196 (28.6%) | 69 (33.3) | 4 (18.2%) |  |
|  | Current | 182 (26.6%) | 42 (20.3%) | 6 (27.3%) |  |
| Body mass index | | 21.8 ± 3.4 | 21.4 ± 3.9 | 19.8 ± 5.1 | 0.018 |
| Previous TB | | 104 (15.2%) | 34 (16.3%) | 6 (27.3%) | 0.302 |
| Comorbidity | |  |  |  |  |
|  | Diabetes | 99 (24.8%) | 34 (27.4%) | 5 (41.7%) | 0.372 |
|  | Malignancy | 17 (4.2%) | 5 (4.0%) | 0 (0.0%) | 0.765 |
|  | Renal disease | 3 (0.8%) | 1 (0.8%) | 0 (0.0%) | 0.953 |
|  | Gastric surgery | 2 (0.5%) | 1 (0.8%) | 0 (0.0%) | 0.892 |
|  | Liver cirrhosis | 3 (0.8%) | 2 (1.6%) | 0 (0.0%) | 0.645 |
|  | COPD | 9 (2.2%) | 3 (2.4%) | 0 (0.0%) | 0.864 |
|  | HTN | 80 (20.0%) | 20 (16.1%) | 1 (8.3%) | 0.404 |
|  | BPH | 5 (1.2%) | 3 (2.4%) | 0 (0.0%) | 0.587 |
| **Laboratory findings** | |  |  |  |  |
| WBC, μL*1000 |  | 6.4 [5.1, 8.1] | 6.3 [5.2, 7.9] | 5.9 [45, 7.8] | 0.435 |
| Neutrophil (%) | | 62 [54, 70] | 64 [53, 71] | 63 [52, 71] | 0.725 |
| Lymphocyte (%) | | 25.1 ± 10.9 | 25.4 ± 11.3 | 27.8 ± 13.1 | 0.568 |
| Hb, g/dL |  | 13.0 ± 2.0 | 13.0 ± 1.7 | 12.8 ± 1.8 | 0.839 |
| Platelet, μL*1000 |  | 268.4 ± 95.8 | 270.9 ± 103.0 | 240.9 ± 119.0 | 0.410 |
| Albumin, g/dL |  | 4.1 [3.7, 4.4] | 4.1 [3.6, 4.4] | 4.1 [3.9, 4.3] | 0.691 |
| Protein. g/dL | | 7.2 [6.8, 7.7] | 7.2 [6.7, 7.6] | 7.4 [7.1, 7.9] | 0.407 |
| AST, U/L |  | 24 [19, 32] | 25 [19, 32] | 31 [21.8, 47.3] | 0.015 |
| ALT, U/L |  | 18 [12, 26] | 19 [13, 28] | 18.5 [13, 30.3] | 0.669 |
| **Radiographic feature** | |  |  |  |  |
| Cavity |  | 52 (7.6%) | 29 (13.9%) | 1 (4.5%) | 0.015 |
| **Adverse event** |  |  |  |  |  |
| Any |  | 135 (19.6%) | 38 (18.2%) | 3 (13.6%) | 0.270 |
| Hepatotoxicity |  | 47 (6.8%) | 12 (5.7%) | 0 (0%) | 0.394 |
| Skin rash |  | 26 (3.8%) | 10 (4.8%) | 0 (0%) | 0.509 |
| GI trouble |  | 40 (5.8%) | 16 (7.7%) | 2 (9.1%) | 0.545 |
| CBC abnormalities |  | 10 (1.5%) | 3 (1.4%) | 0 (0%) | 0.851 |
| Fever |  | 3 (0.4%) | 2 (1.0%) | 0 (0%) | 0.672 |
| Arthralgia |  | 12 (1.7%) | 5 (2.4%) | 0 (0%) | 0.62 |
| Neuropathy |  | 5 (0.7%) | 3 (1.4%) | 0 (0%) | 0.568 |

Abbreviations: TB, tuberculosis; NTM, non-tuberculosis mycobacterium; LTBI, latent tuberculosis infection; COPD, chronic obstructive pulmonary disease; HTN, hypertension; BPH, benign prostate hyperplasia; WBC, white blood cell count; Hb, hemoglobin; Na, sodium; K, potassium, BUN, blood urea nitrogen; Cr, creatinine; AST, aspartate aminotransferase; ALT, alkaline aminotransferase; CBC, complete blood count

**Supplementary Figure S1**. Flow chart of study population selection


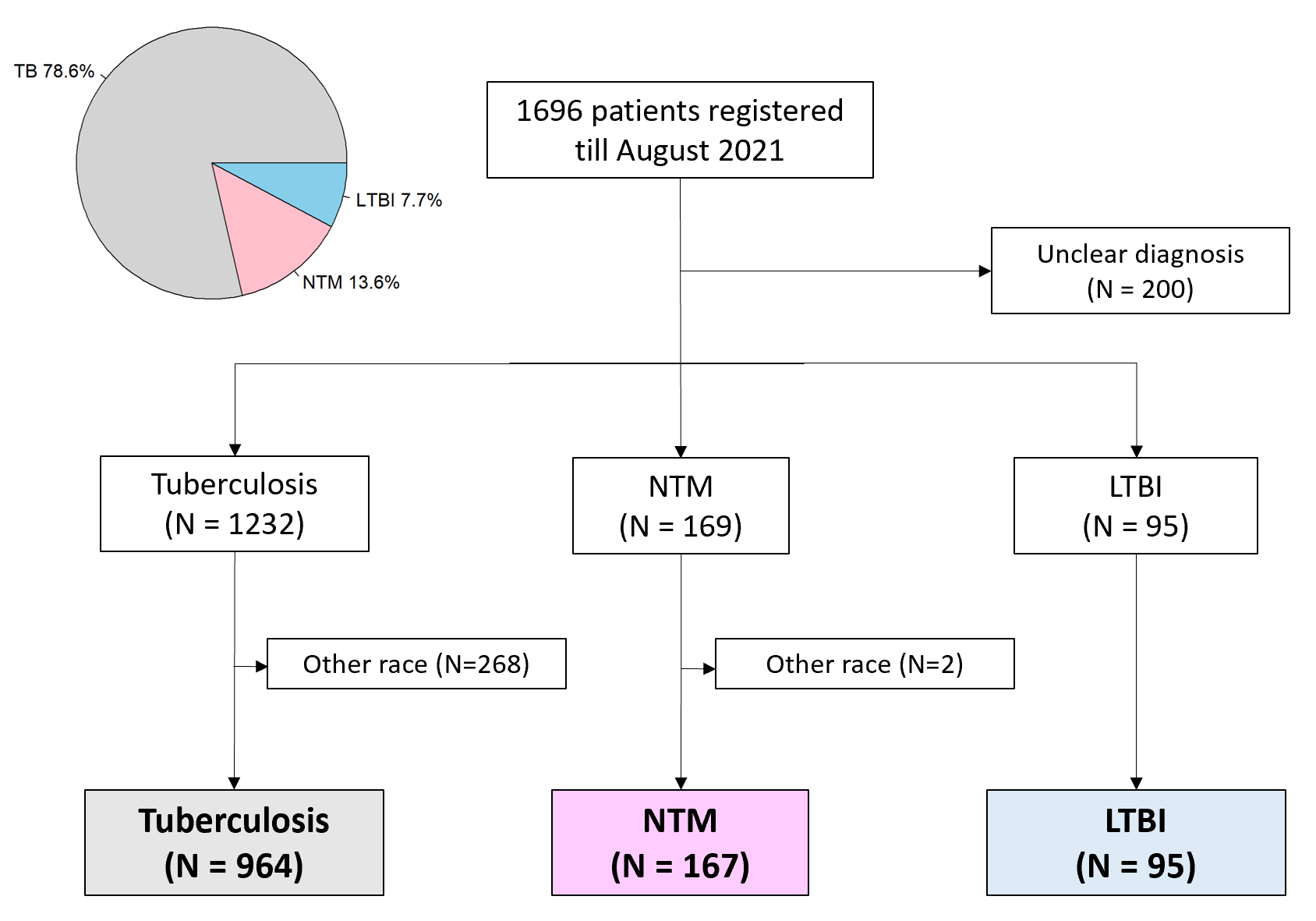


Abbreviations: TB, tuberculosis; NTM, non-tuberculosis Mycobacterium; LTBI, latent tuberculosis infection

**Supplementary Figure S2**. Age group distribution of (A) tuberculosis, (B) non-tuberculosis Mycobacterium, and (C) latent tuberculosis infection, stratified by sex


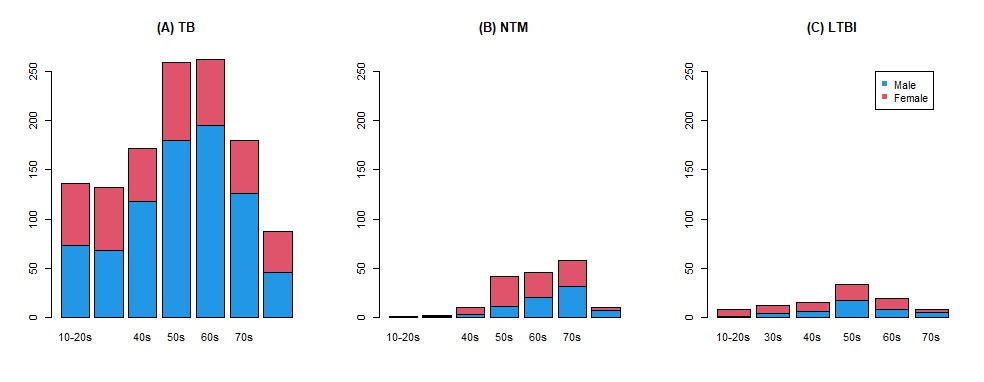
 Abbreviations: TB, tuberculosis; NTM, non-tuberculosis Mycobacterium; LTBI, latent tuberculosis infection

**Supplemental Figure S3**. The results of LASSO regression analysis of LASSO coefficient profiles and selection of in the LASSO regression analysis for (A) TB vs. NTM, (B) TB vs. LTBI, and (C) NTM vs. LTBI

(A) TB vs NTM


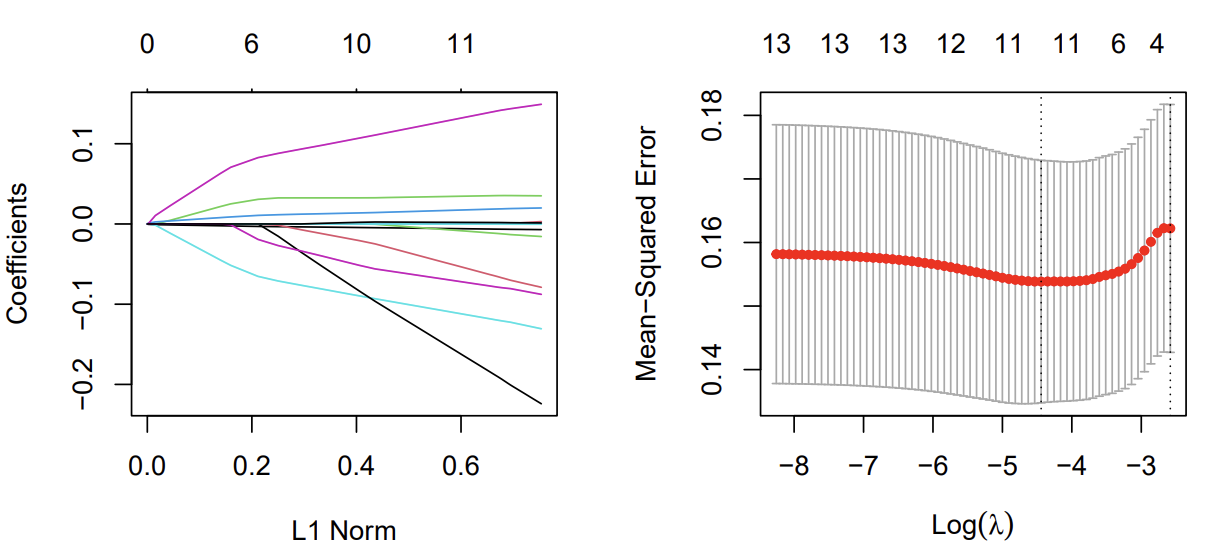


(B) TB vs. LTBI


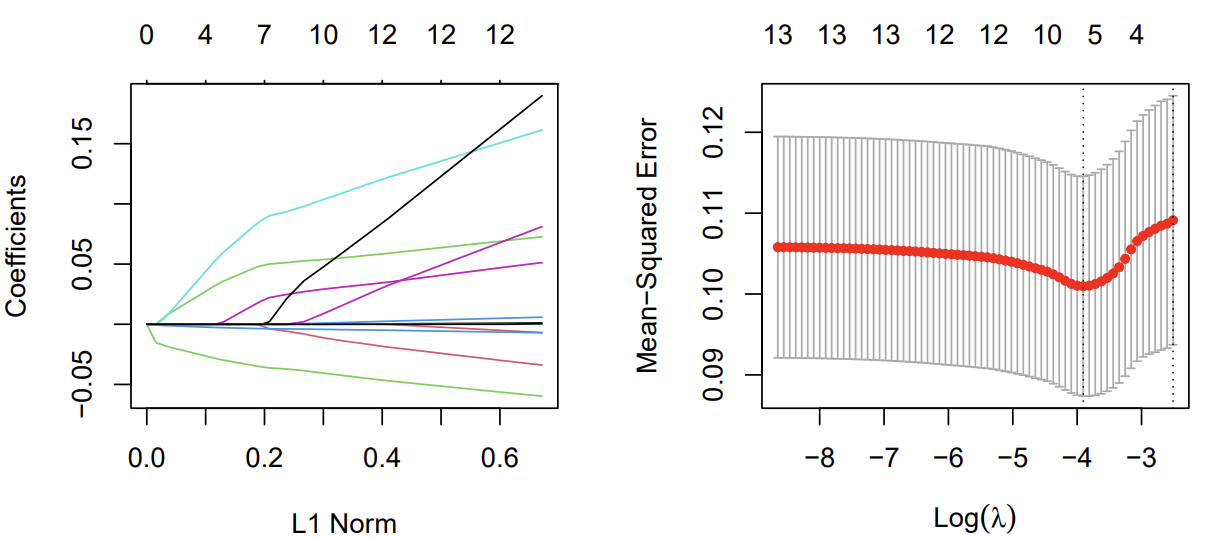


(C) NTM vs. LTBI


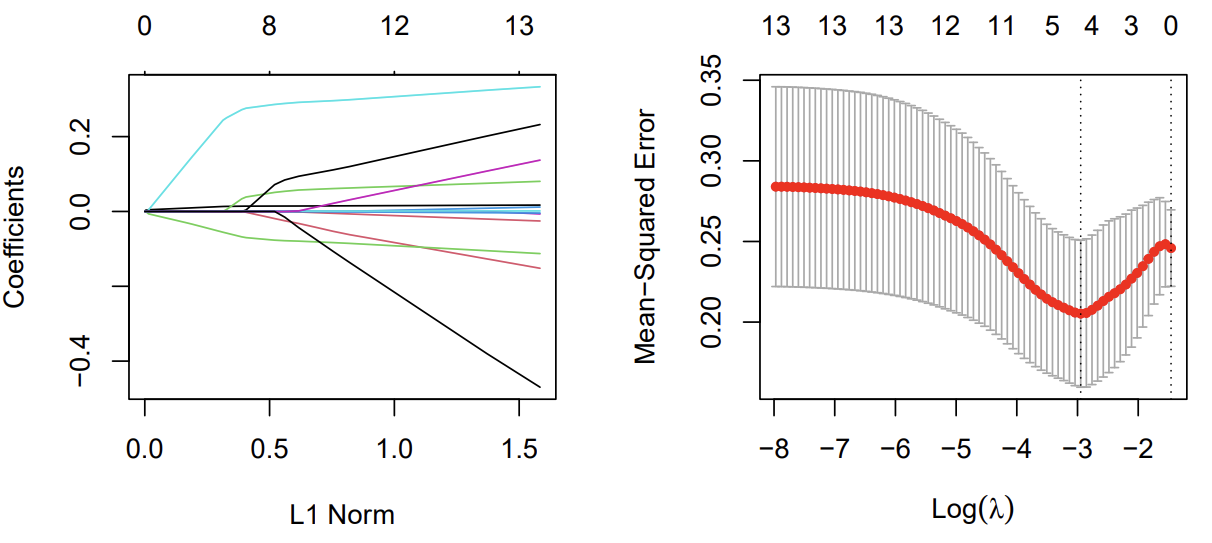


The dotted vertical lines are plotted at the optimal values following the minimum criteria (right) and “one standard error” criteria (left)

**Supplementary Figure S4**. Receiver operating characteristic curve for prediction models


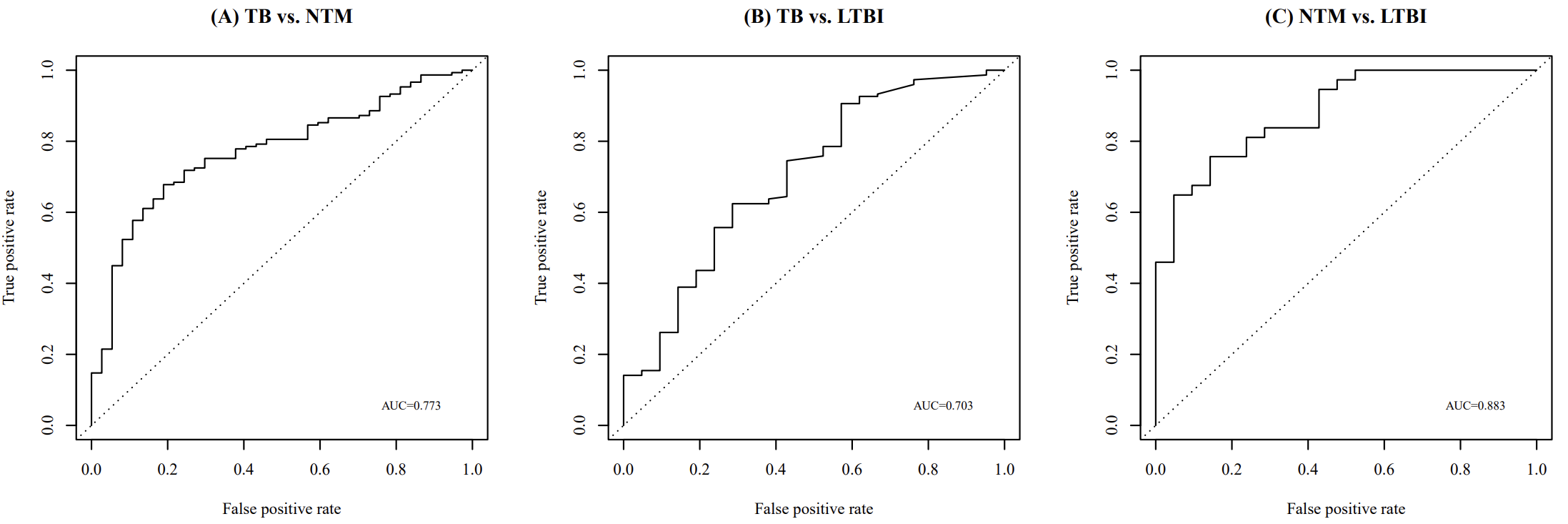


Abbreviations: TB, tuberculosis; NTM, non-tuberculosis Mycobacterium; LTBI, latent tuberculosis infection

**Supplemental Figure S5**. Receiver operating characteristic curve for the (A) NAT2 and (B) SLCO1B1 phenotypes to predict hepatotoxicity


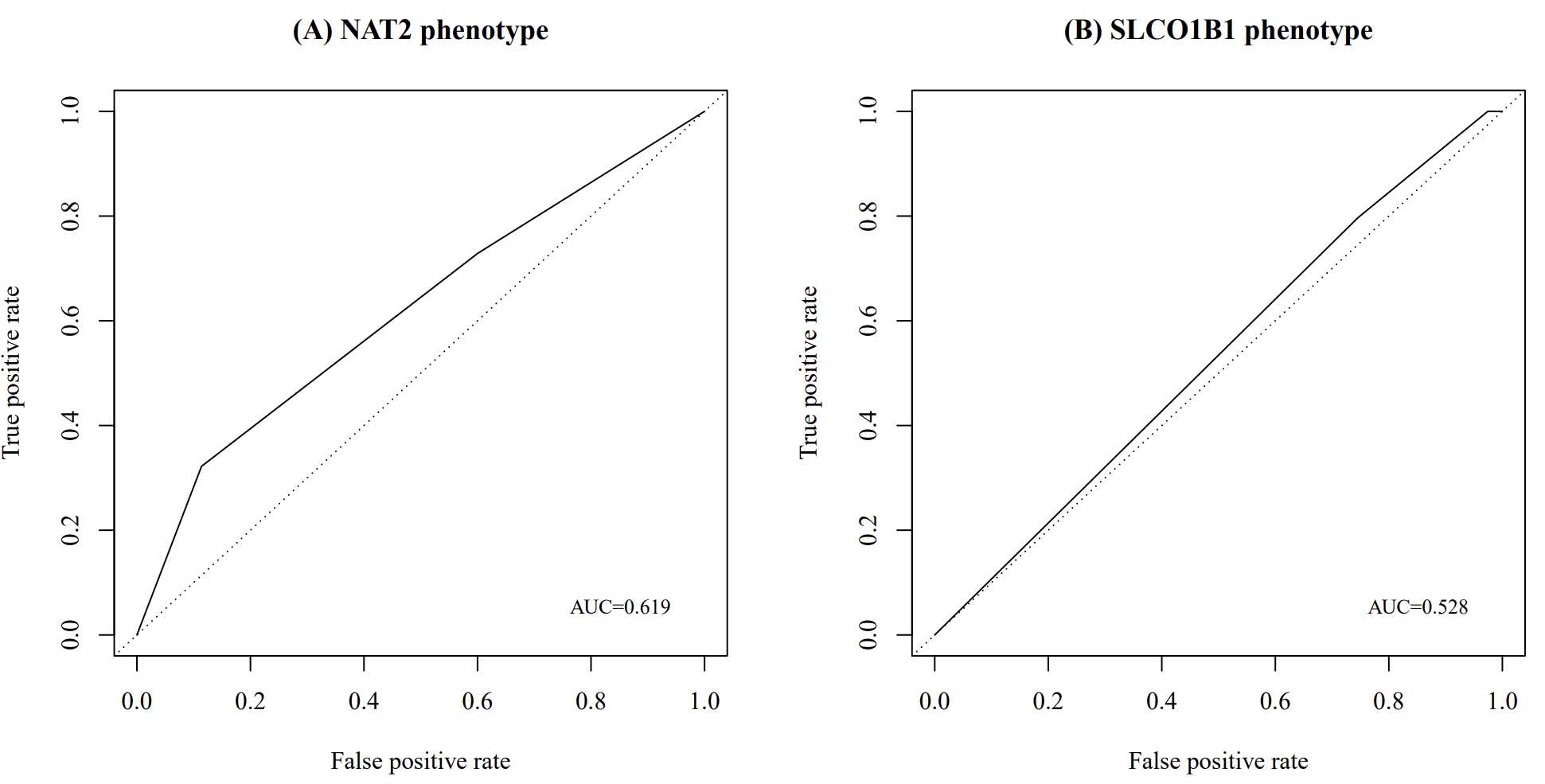

Supplement: Supplementary file 1 — Additional file 1. [file 12890_2023_2646_MOESM1_ESM.docx]
